# Neutrino and EM asterometric detection of habitable exoplanets

**DOI:** 10.6026/97320630019235

**Published:** 2023-03-31

**Authors:** Paul Shapshak

**Affiliations:** 1 Global Disease Institute, Tampa General Hospital, Department of Internal Medicine, Morsani College of Medicine, University of South Florida, Tampa, Florida 33606, USA

**Keywords:** Neutrino, EM asterometric, black holes, neutron, dwarf stars

## Abstract

Discovering habitable exoplanets and exomoons increases the possibility of detecting extraterrestrial life. A bilateral approach, using neutrino and electromagnetic (EM) radiation technologies, can be used to simultaneously characterize star types that generally have exoplanets and exomoons. This includes cool main-sequence, sub-giant, and red-giant stars. Additionally, supernovae, black holes, and neutron and dwarf stars, will be included to widen the investigation, since they sometimes have companions, including stars and planets. Currently, space exploration is advancing beyond the solar system and proliferating into deep space. For this expansion, sophisticated artificial intelligence (AI) is required and being developed for self-coordination and interactive regulation of the various exploratory vehicles and telescopes. [1,2, 3,4,5, 6]

## Purpose:

Future stellar system research could synchronously utilize detectors for neutrinos and EM (X-ray, visible, and infra-red radiation.) Each of these methodologies detects different target properties. These properties include physical and chemical properties, evolution, and origin of target host star systems and their habitable exoplanets and exomoons. Favorable conditions and signatures of life may be detected. Incidental to the discovery and possible existence of astrobiology, astrovirology, and astromicrobiology elsewhere in the universe, detection of extra-terrestrial intelligence (ETI) may be possible. [7, 8,9,10]

## Rationale:

Over the millenia, the possible existence of life elsewhere in the universe has been of central and enigmatic concern. However, the universe is more habitable than previously considered. As of February 22nd, 2023, 6,176 candidate exoplanets were detected, 5,272 were confirmed, and 63 habitable exoplanets were identified. This is a remarkable achievement, and further endeavors and discoveries are forthcoming. Concomitant uses of the two technologies, neutrinos and EM, improve reliability of the endeavor. Using both study windows promotes consistent production of confirmatory and reliable data. In this respect, usage of prospective study designs produces results with greater statistical significance, compared to serendipitous studies that were not prospectively designed. [3, 4,5,6, 7,8,9, 10,11]

EM radiation is the primary means of asterometric exploration and communication. EM signals may be perturbed by interstellar and intergalactic distance and occlusion. The use of neutrinos complements such studies as neutrinos also emanate from distant targets. Neutrinos have a high penetrance, which allows them to traverse inter-galactic distances. Neutrinos from stellar sources (e.g. supernovae) reach the Earth prior to photons from the same sources, due to the high penetrance of neutrinos. However, neutrino studies are still at an early stage of development compared to EM studies and are being optimized for asterometric analysis. Using the two methodologies in parallel increases the sensitivity and probability of producing signatures related to astrobiology and ETI detection. This has additional significance for scenarios under which humans may actually encounter exobiologies, exovirologies, and exomicrobiologies. Various safety and survival issues ensue that have been previously discussed. [10, 12,13]

## Difficulties:

Problems in the use of neutrinos include issues of the current huge sizes of transmitter and receiver apparatus, costs, detection efficiency, specificity, lack of focal point source definition, and improving understanding of target dynamics. The development and evolution of AI to control machines for expansion into deep space is ongoing. A long-range time scale is projected as factories and assembly sites must be constructed on the Moon or Mars, and in inter-planetary space in the Solar system, if resources permit. Additional difficulties include improving engineering focused directional long-distance beams, sensitivity, resolution, and signal purity of transmitted and received neutrinos. Signal sensitivity and specificity of source locations are also problems. Clearly, miniaturization of these machines will facilitate development. [6, 13, 14,15,16]

## Background:

Frank Drake, Carl Sagan, William Newman, Freeman Dyson, Arthur Clark, and colleagues developed signposts of technological civilization development. They deduced 20th century technological development as a relatively early stage of space exploration. Thus, now in the 21st century, radiotelescope satellite programs are increasing the sophistication and complexity of space exploration. EM space telescope observatories include Hubble, Kepler, Fermi gamma, Spitzer, Dark Universe, and recently, Transiting Exoplanet Survey Satellite (TESS) Asteroseismic Science Consortium (TASC) space telescope. Neutrino detectors include Super-Kamiokande, Borexino, Antares, Sudbury Neutrino observatory, Daya Bay Reactor Neutrino Experiment, Germanium detector array, Kamioka Liquid Scintillation Antineutrino detector, KM3NET, Cherenkov detector, Gallex, Katrin, Scintillator, Dumand Project, and Ino Peak Project Theni. [17,18,19, 20]

## Neutrinos:

Neutrino research is used in many fields including asterometrics, astronomy, astrophysics, and cosmology. Neutrino sources include the Sun, other stars, supernovae, neutron stars, blackholes, cosmic rays, particle accelerators, nuclear reactors, as well as neutrinos from the early universe and big bang. Thus, there is an intense neutrino crisscrossing background, which also includes a temporal dimension of their production since the Big Bang. Neutrinos interact very weakly with matter; however, their detection is greatly enhanced by their huge numbers. Neutrinos undergo flavor oscillations among electron-, mu-, and tau-neutrino states. In particular, neutrino profiles are designed to characterize various star types and their evolution and their association with habitable exoplanets and exomoons. Corollary to these studies, their use is pertinent to detection of ETI. Very large neutrino detectors and detection technologies include the use of Cerenkov radiation, liquid scintillation, iron calorimeter, and liquid Argon. Neutrino detectors are found in many countries including the USA, Canada, Italy, France, Japan, Russia, India, South Korea, China, as well as in the Mediterranean Sea and beneath Antarctica. [14,16, 2121,^22^]

## Communications:

Since 1977, Pasachoff, Saenz, and colleagues proposed the use of neutrinos in communications. This has been accomplished with terrestrial communications and could be used for long-distance interstellar and intergalactic messaging. These considerations are made more complex as neutrino properties are incomplete and not fully understood. For example, neutrino analyses indicate neutrinos have mass, in contradiction to the Standard Model of particle physics, and that consequently, they undergo flavor oscillations as do quarks. Moreover, do neutrinos obey Pauli exclusion statistics, Fermi statisticsm or Bose-Einstein condensate statistics? Are light neutrinos components of Dark Matter? Are neutrinos Dirac or Majorana? Is there a double beta decay model where Aá A' + 2 anti-neutrinos + 2 electrons? ("A" is any of twelve elements, which may support double beta-decay, including, for example, calcium-48, selenium-82, tellurium-128 and tellurium-130.) Such fundamental research questions must be solved, prior to commencing reliable detection of neutrino communications from prospective candidate ETIs, as well as improving human technology development. [10, 13,23,24, 25,26]

## Conclusions:

Neutrino and EM detection systems are best used synchronously and prospectively to detect and analyze stars and their habitable exoplanets and exomoons. This is taking place during space exploration development and expansion. Development of advanced AI-controlled communications and signaling among space probes will be helpful and obligatory. [10, 24,26]
